# Optimising Decision-Making in Child and Adolescent Mental Health Crisis Calls: A Quality Improvement Initiative for Clinicians

**DOI:** 10.1192/j.eurpsy.2026.10678

**Published:** 2026-07-31

**Authors:** T. Mehdi, E. Perrin, A. Caston, A. Daud

**Affiliations:** Child and Adolescent Mental Health, Berkshire Healthcare NHS Foundation Trust, Reading, United Kingdom

## Abstract

**Introduction:**

England’s National Health Service (NHS) Long Term Plan (2019) mandated 24/7, open-access mental health crisis care via NHS 111, with COVID-19 accelerating delivery; by August 2024, NHS 111 provided nationwide, round-the-clock access linking children and young people (CYP) to local crisis teams. Berkshire Healthcare NHS Foundation Trust (BHFT) established a 24-hour Child and Adolescent Mental Health (CAMH) Services crisis line; however, clinicians receiving unplanned calls lacked a structured approach to call management, and internal reviews suggested that around 50% of decisions were inappropriate, highlighting the need for improvement.

**Objectives:**

This Quality Improvement (QI) project aimed to embed a standardised, evidence-informed process for decision-making during and after child and adolescent mental health crisis calls. It sought to provide clinicians with a structured information-gathering framework that preserves therapeutic engagement, strengthen confidence and consistency when agreeing next steps and safety/support plans with young people and families and optimise team resources. The primary aim was to increase the proportion of appropriate decisions to at least 80%.

**Methods:**

We used a QI approach with Plan–Do–Study–Act (PDSA) cycles in the Berkshire Healthcare NHS Foundation Trust CAMH crisis service. Two panels of 3–4 crisis clinicians, each moderated by a senior specialist, reviewed notes from 22 consecutive crisis calls over two months; 50% of call outcomes were judged inappropriate. Root cause analysis identified insufficient information gathering and the absence of a clear call structure as the predominant contributors. Achievable countermeasures were co-designed and implemented, which included the development of a crisis call triage guide; followed by a repeat review of 22 crisis calls over a subsequent two-month period to assess impact.

**Results:**

After implementation, 21/22 crisis-call outcomes (95%) were rated appropriate, up from 50% at baseline. Clinicians reported the guide was universally helpful, increased confidence, clarified outcome pathways, and enabled timely escalation to senior clinicians when uncertain. Decision-making shifted from risk-centric assessments to a formulation- and needs-led approach.

**Image 1:**
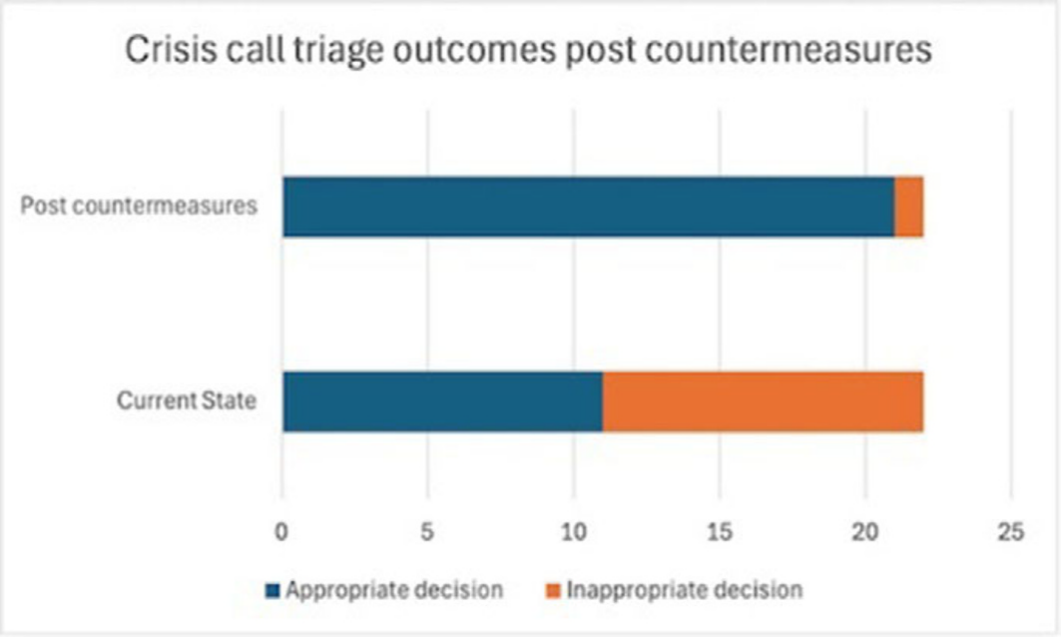

**Image 2:**
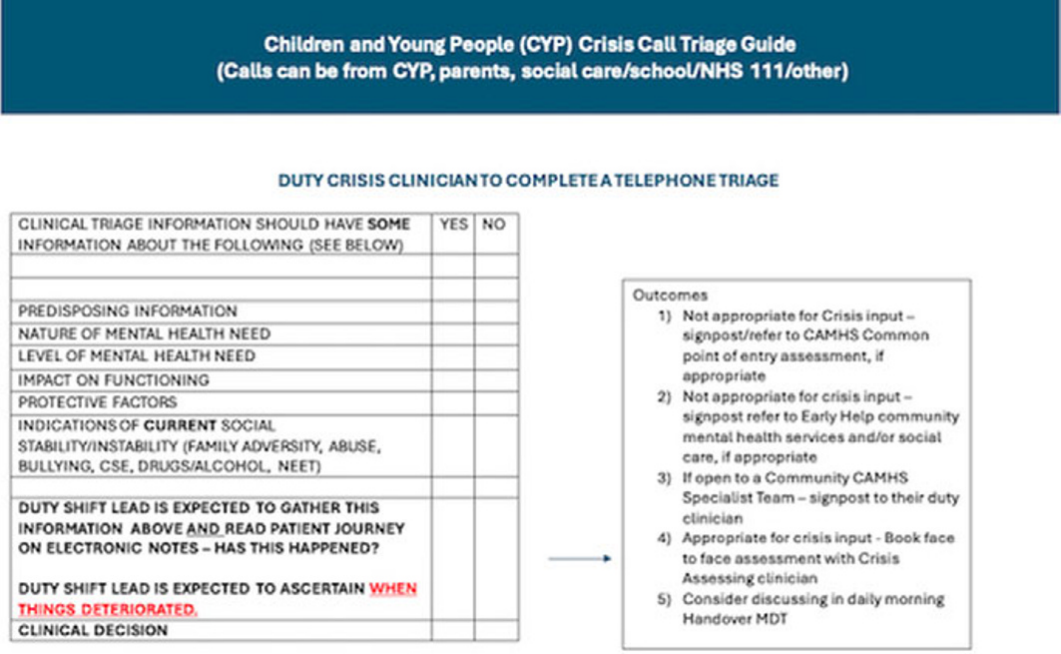
Image 2: Long description.

**Image 3:**
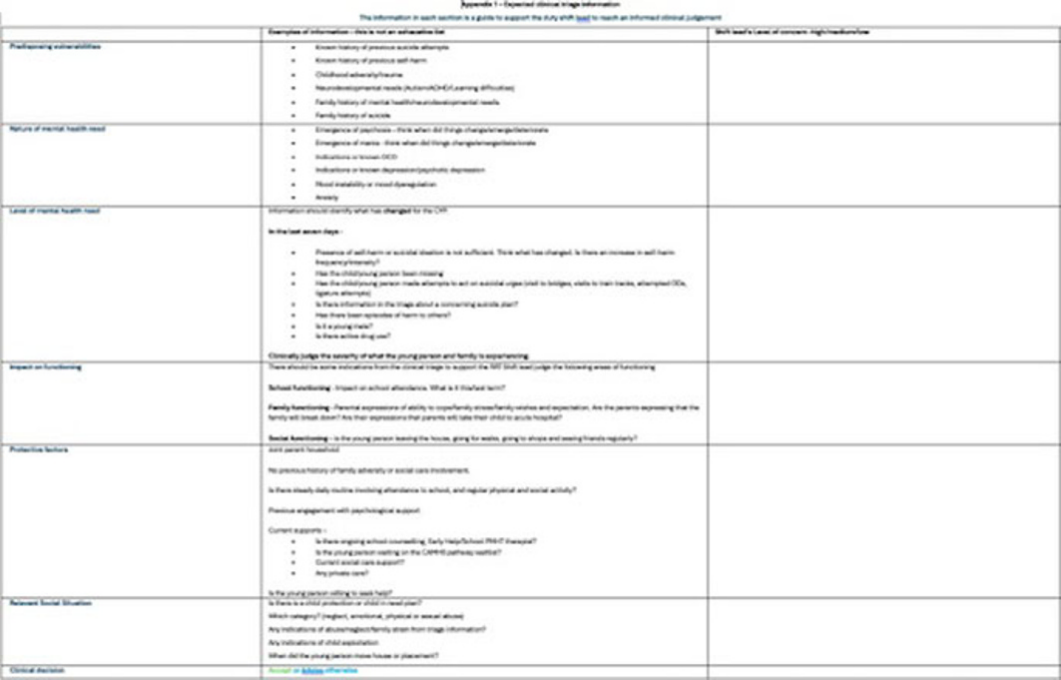
Image 3: Long description.

**Conclusions:**

Co-produced and evidence-informed, the clinician crisis call triage guide—implemented within a QI framework—markedly improved crisis-call decisions (95% appropriate vs 50% baseline), shifted practice from risk-centric to needs-led, formulation-based care, and increased clinician confidence, clarity on next steps, and timely senior escalation. Clinicians reported that the crisis call triage guide offered a simple and standardised call structure. Despite limitations (single service, modest sample, short follow-up), the approach is feasible and scalable.

**Disclosure of Interest:**

None Declared

## Long descriptions

### Image 2: Long description

A flowchart and checklist titled Children and Young People C Y P Crisis Call Triage Guide.

At the top, a blue banner states that calls can be from C Y P, parents, social care, school, N H S 1 1 1, or others. Below this, the instruction reads DUTY CRISIS CLINICIAN TO COMPLETE A TELEPHONE TRIAGE.

The left side contains a table with three columns: Clinical Triage Information, Yes, and No. The rows include:

* PREDISPOSING INFORMATION

* NATURE OF MENTAL HEALTH NEED

* LEVEL OF MENTAL HEALTH NEED

* IMPACT ON FUNCTIONING

* PROTECTIVE FACTORS

* INDICATIONS OF CURRENT SOCIAL STABILITY/INSTABILITY (FAMILY ADVERSITY, ABUSE, BULLYING, C S E, DRUGS/ALCOHOL, N E E T)

* DUTY SHIFT LEAD IS EXPECTED TO GATHER THIS INFORMATION ABOVE AND READ PATIENT JOURNEY ON ELECTRONIC NOTES - HAS THIS HAPPENED?

* DUTY SHIFT LEAD IS EXPECTED TO ASCERTAIN WHEN THINGS DETERIORATED (text in red)

* CLINICAL DECISION

A blue arrow points from the table to a box on the right titled Outcomes, which lists five numbered points:

1. Not appropriate for Crisis input - signpost/refer to C A M H S Common point of entry assessment, if appropriate.

2. Not appropriate for crisis input - signpost refer to Early Help community mental health services and/or social care, if appropriate.

3. If open to a Community C A M H S Specialist Team - signpost to their duty clinician.

4. Appropriate for crisis input - Book face to face assessment with Crisis Assessing clinician.

5. Consider discussing in daily morning Handover M D T.

Navigate back to Image 2:.

### Image 3: Long description

The table consists of three columns: Category, Examples of information, and Shift lead's Level of concern (high/medium/low).

Row 1: Predisposing vulnerabilities. Examples include known history of previous suicide attempts or self-harm, childhood adversity/trauma, neurodevelopmental needs (Autism/A D H D/Learning difficulties), and family history of mental health/neurodevelopmental needs or suicide.

Row 2: Nature of mental health need. Examples include emergence of psychosis or mania, indications of known O C D, depression/psychotic depression, mood instability, or anxiety.

Row 3: Level of mental health need. Focuses on what has changed in the last seven days, such as increased self-harm frequency, missing person reports, suicidal urges, specific suicide plans, harm to others, or active drug use.

Row 4: Impact on functioning. Evaluates school functioning (attendance), family functioning (ability to cope), and social functioning (leaving the house/seeing friends).

Row 5: Protective factors. Includes joint parent household, no previous social care involvement, steady daily routine, previous psychological support, and current supports like school counseling or C A M H S pathway waiting lists.

Row 6: Relevant Social Situation. Checks for child protection plans, categories of abuse, family strain, child exploitation, or recent housing changes.

Row 7: Clinical decision. Options are Accept or Advise otherwise.

Navigate back to Image 3:.

